# Biopsychosocial effects and experience of use of robotic and virtual reality devices in neuromotor rehabilitation: A study protocol

**DOI:** 10.1371/journal.pone.0282925

**Published:** 2023-03-10

**Authors:** Francesco Zanatta, Patrizia Steca, Cira Fundarò, Anna Giardini, Guido Felicetti, Monica Panigazzi, Giovanni Arbasi, Cesare Grilli, Marco D’Addario, Antonia Pierobon

**Affiliations:** 1 Department of Psychology, University of Milano-Bicocca, Milan, Italy; 2 Istituti Clinici Scientifici Maugeri IRCCS, Neurophysiopathology Unit of Montescano Institute, Montescano, Italy; 3 Istituti Clinici Scientifici Maugeri IRCCS, Information Technology Department of Pavia Institute, Pavia, Italy; 4 Istituti Clinici Scientifici Maugeri IRCCS, Neuromotor Rehabilitation Unit of Montescano Institute, Montescano, Italy; 5 Istituti Clinici Scientifici Maugeri IRCCS, Occupational Physiatry and Ergonomics Unit of Montescano Institute, Montescano, Italy; 6 Istituti Clinici Scientifici Maugeri IRCCS, Psychology Unit of Montescano Institute, Montescano, Italy; Prince Sattam Bin Abdulaziz University, College of Applied Medical Sciences, SAUDI ARABIA

## Abstract

**Background:**

Robot-assisted therapy (RAT) and virtual reality (VR)-based neuromotor rehabilitation have shown promising evidence in terms of patient’s neuromotor recovery, so far. However, still little is known on the perceived experience of use of robotic and VR devices and the related psychosocial impact. The present study outlines a study protocol aiming to investigate the biopsychosocial effects and the experience of use of robotic and non-immersive VR devices in patients undergoing neuromotor rehabilitation.

**Methods:**

Adopting a prospective, two-arm, non-randomized study design, patients with different neuromotor diseases (i.e., acquired brain injury, Parkinson’s Disease, and total knee/hip arthroplasty) undergoing rehabilitation will be included. In a real-world clinical setting, short- (4 weeks) and long-term (6 months) changes in multiple patient’s health domains will be investigated, including the functional status (i.e., motor functioning, ADLs, risk of falls), cognitive functioning (i.e., attention and executive functions), physical and mental health-related quality of life (HRQoL), and the psychological status (i.e., anxiety and depression, quality of life satisfaction). At post-intervention, the overall rehabilitation experience, the psychosocial impact of the robotic and VR devices will be assessed, and technology perceived usability and experience of use will be evaluated through a mixed-methods approach, including both patients’ and physiotherapists’ perspectives. Repeated measures within-between interaction effects will be estimated, and association analyses will be performed to explore the inter-relationships among the variables investigated. Data collection is currently ongoing.

**Implications:**

The biopsychosocial framework adopted will contribute to expanding the perspective on patient’s recovery within the technology-based rehabilitation field beyond motor improvement. Moreover, the investigation of devices experience of use and usability will provide further insight into technology deployment in neuromotor rehabilitation programs, thereby maximising therapy engagement and effectiveness.

**Trial registration:**

ClinicalTrials.gov ID: NCT05399043.

## 1. Background

Over the past two decades, due to the aging population and the consequent increase in related diseases, serious healthcare challenges have emerged in most countries. This has generated the need for urgent solutions, including the implementation of interdisciplinary and innovative approaches along with technology-enabled smart healthcare strategies [1]. Contextually, the field of neuromotor rehabilitation has shown increasing interest in the introduction of robotic and virtual reality (VR) technologies given their multipurpose application to the patient recovery process [2, 3].

Robot-assisted therapy (RAT) has reported promising evidence so far, showing several advantages such as the possibility to provide repetitive, intensive, and task-oriented rehabilitation activities, as well as the opportunity to implement a smaller workforce, optimized exercise, and real-time quantitative motor impairment assessment and monitoring. Furthermore, the use of robotic devices has benefited from more complex and customized interventions, by managing the parameters that allow for the personalization of the recovery program based on patient characteristics, and by increasing the amount and quality of the treatment that can be administered [4]. Essentially, robotic devices have been applied to the rehabilitation process according to the principles of motor learning and neuroplasticity with the aim of better maximizing afferent input from peripheral joints and supply task-specific stimulation to the central nervous system so as to promote functional recovery of both the upper and lower limbs. For the former, the RAT consists in the execution of task-specific exercises with different levels of intensity and technology-based assistance promoting motor relearning of motor function, including arm and/or extremity range of motion, strength, and functional flexibility. For the lower limbs, the robot-assisted gait training (RAGT) has been widely implemented to target the regaining and the improvement of patient’s mobility, walking ability, and balance. For this purpose, different types of robotic devices (i.e., exoskeletons, end-effectors, soft-robots) have so far been implemented to target diverse chronic and complex diseases (of a traumatic, vascular or neurodegenerative nature) like stroke [5, 6], traumatic brain injury (TBI) and spinal cord injury (SCI) [7, 8], multiple sclerosis (MS) [9, 10], and Parkinson’s disease (PD) [11, 12], reporting evidence in favor of their feasibility and an improvement in patient autonomy in the activities of daily living (ADLs) and health-related quality of life (HRQoL). Moreover, prior research has also observed, beyond motor improvement, the psychological impact of implementing this technology, reporting for example significant post-intervention changes in patients’ depression and anxiety symptoms along with increased well-being [13, 14]. Despite the significance of these findings, studies including psychological factors as primary outcomes are still scant. Further research is needed on the effects of RAT adopting a psychosocial perspective, especially considering the well-known importance of the perception of disability in chronic neurological populations.

Likewise, VR technology has been shown to be a promising tool to improve rehabilitation outcomes. When various technical devices (e.g., head-mounted displays, desktop computers, motion capture and tracking systems, motion-sensing gloves) are implemented, it can deliver, through different levels of immersion, realistic experiences by the creation of and interaction with virtual environments (VEs) closely resembling everyday environments [15]. To date, a wide range of studies have tested and demonstrated the efficacy, for example, of VR-based treadmill training for lower extremities to reduce risk of falls, improve gait and balance [16] or of exercises in reaching and grasping virtual objects for upper extremities to increase arm and manual movement, dexterity, and coordination [17]. Moreover, commercially available serious game platforms (e.g., Nintendo Wii, Microsoft Kinect) have been effectively implemented to combine rehabilitation with the possibility to play games and perform various activities [18, 19]. Exercises through video games (i.e., exergame) have gained increased interest as they provide several advantages when compared to the conventional ones, including the motivation to practice through an interactive and attractive way, the possibility to train motor and cognitive skills performing dual tasks, and the shift of the focus to the outcome of the movement, displayed in the game, from the movement itself. Overall, the efficiency of VR technology may be firstly explained by its peculiarity to provide realistic, meaningful, and repetitive experiences delivered through specific tasks whose difficulty and complexity can be adapted according to each patient’s needs and characteristics [20]. Secondly, VR systems can also supply real-time and goal-directed feedback, which are considered key components facilitating quick self-correction and accommodating motor relearning [21, 22]. Therefore, significant improvements in motor impairment have so far been observed across pathologies. Nevertheless, as VR-based interventions are increasingly used, mixed results are reported in the literature. On the one hand, this heterogeneity is mainly due to the large number of technological platforms implemented, which implies a possible moderation effect of the VR technology-related variables (e.g., level of immersion, customization of the systems). On the other hand, it may be influenced by the characteristics of the population included (e.g., age, clinical diagnosis, disease severity) [3]. In general, the multimodal and multisensory stimulation as well as the possibility of personalization offered by VR have been shown to have the potential to increase patient engagement throughout rehabilitation programs, ultimately improving treatment compliance [23, 24]. Beside this advantage, this stimulation and complexity have been shown to be determinant in obtaining significant improvements in cognitive outcomes (e.g., memory, dual tasking, and visual attention) and secondarily in psychological functioning (e.g., anxiety reduction, higher well-being, and increased use of coping strategies) [25].

Consistent promising evidence has also been reported in the studies coupling VR to RAT. A recent study describing the effects of adopting this therapeutic approach has for example evidenced a significant improvement in HRQoL in terms of perceived mental and physical state, and in different cognitive domains, like cognitive flexibility and shifting abilities, selective attention and visual search [26]. Again, another work highlighted the positive impact of combining the two technologies showing significant changes in patient’s functionality, mobility, static and dynamic balance, and in problem-solving abilities [27]. Overall, the integration of VR in RATs has so far showed encouraging results. Even though robots are usually well accepted by patients, RAT may be perceived as poor motivating. Adding VR may overcome this limit as it was shown to augment the motivation to endure practice and to strengthen performance awareness, as well as to stimulate visual, tactile and auditory input, ultimately inducing, at synaptic level, deep cortical and subcortical changes that are central for motor relearning [28].

Ensuring adequate patient engagement and adherence to treatment represents another challenge to maximize therapy efficacy and success. Accordingly, the introduction of technological devices in rehabilitation programs has raised the issue of device usability and the related experience of use, with the idea that the more positively the technology is perceived and accepted, the stronger the patient’s motivation and satisfaction with the program and, thus, the more effective the therapy [29]. Usability refers to a patient’s perception and ability to use a device to achieve goals effectively, efficiently, and satisfactorily [30]. As a result, factors like device acceptability, usefulness, ease-of-use, and learnability are particularly important. These factors, however, do not always sufficiently explain the complexity of the experience of use. To better develop and expand the concept of device use, further aspects that embrace the full subjective user experience need to be considered too. Of these, socio-cognitive and experiential factors (e.g., emotions, expectations, motivation, satisfaction) may play a pivotal role in determining rehabilitation outcomes. For instance, the degree of a perceived sense of presence or the intensity of the adverse effects related to the exposure to VEs may deeply impact the quality of therapy delivery and, as a result, treatment outcomes, likewise the perceived safety and level of comfort in relation to the use of a robotic device [31, 32]. For the reasons above, exploring these indicators should be addressed, taking into account patients’ perspectives. In particular, when aiming to understand a patient’s perception and attitude toward the use of a device, it is likewise essential to consider the context in which it is used. In most cases, the implementation of technology during the rehabilitation program is necessarily supported and mediated by a therapist, whose expectations and views may be transferred to the patient [33, 34]. Accordingly, the social influence exerted by healthcare professionals represents an additional factor that should be considered as a possible determinant of the quality of patients’ experience of use.

Although the literature has already shown promising evidence in favor of the application of robotics and VR to rehabilitation programs, this research field is relatively recent. Therefore, beside the need for further studies corroborating the potential of this technology in terms of efficacy, more effort is needed to understand the related multidimensional impact across clinical populations by considering the centrality of factors like perceived HRQoL, and psychological and cognitive functioning. Accordingly, recent evidence has highlighted the adoption of a holistic approach in the field of technology-based neurorehabilitation as a still open challenge, where multiple patient’s health domains and their complementarity should be considered beyond motor improvement [27, 35, 36]. Likewise, other recent studies encouraged research on this topic to include formal evaluations with respect to the technology characteristics, especially to how these are perceived by the users, in order to maximise their functionality and the consequent effectiveness [37, 38]. Following this line, future research should therefore not only address the wider effects of technology implementation in rehabilitation programs, thereby providing clearer evidence on its efficacy and effectiveness, but it should also focus on the experiential factors and the usability issues related to the technology use, facilitating the related scaling-up process.

Based on the background described, it must be overall acknowledged that the wider impact of technology-based rehabilitation on patient’s non-motor outcomes has so far insufficiently investigated and that a consensus on technology formal evaluation procedures within recovery programs still lacks. Deeper investigation and clearer empirical evidence on this topic are therefore required. Accordingly, this paper aims to describe a study protocol called “Perception of High Technology in Rehabilitation: a prospective real-life Study on usability, effectiveness, and health-related quality of life” (PHTinRehab Study), whose main purpose is to explore, in a real-world clinical setting, the experience of use and the biopsychosocial effectiveness of robotics and VR devices in patients undergoing neuromotor rehabilitation. More specifically, the aims are:

- to explore pre- post-treatment differences and long-term effects concerning: patients’ HRQoL, anxiety and depression symptoms, cognitive functioning, and functional status;
- to measure patients’ perceived experience of the rehabilitation program, as well as the perceived psychosocial impact and experience of using the robotic and VR devices (user experience and device usability);
- to observe the differences on technology evaluation, in relation to the pathology, disease severity, and type of device implemented;
- to extend the evaluation of the device perceived usability to the therapists and explore to what extent this is associated to patients’ perception of device usability and overall rehabilitation experience.

These aims present crucial implications for clinical practice and research. The evidence on short- and long-term biopsychosocial effectiveness of technology not only may help to discuss more deeply the impact of RAT and VR on motor improvement, but it would also add knowledge to the effects on patient’s psychological profile, ultimately expanding the perspective on patient recovery. Moreover, exploring patients’ perception of their technology-based rehabilitation program would provide crucial information on how the experience of using the technology devices throughout the recovery may differ depending on the pathology and disease severity. In a personalized medicine perspective, this would help future clinical practice and research to provide validated rehabilitation pathways that are tailored on patients’ needs and characteristics. Moreover, eliciting technology strengths and limitations from the therapists’ perspective would allow to collect informative data from a professional point of view and, thus, provide helpful indications for future devices development or adaptation, ultimately facilitating optimized technology deployment to neuromotor recovery programs.

## 2. Materials and methods

**Fig 1:**
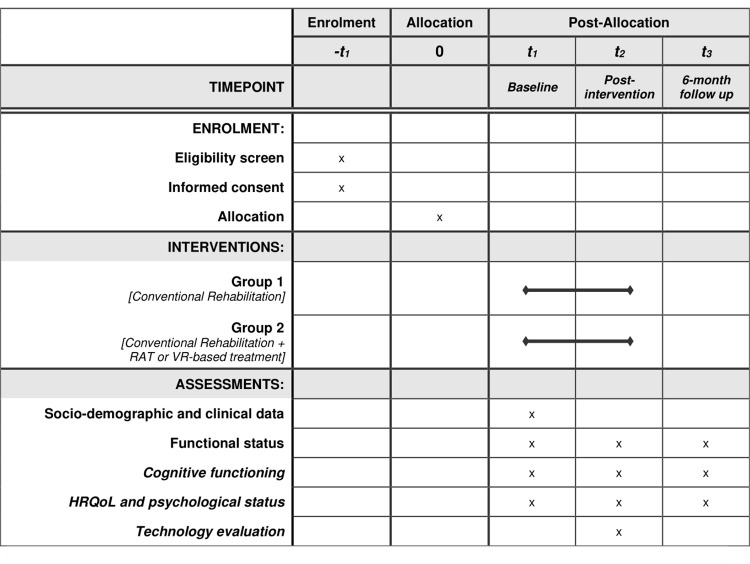
SPIRIT schedule of enrolment, interventions, and assessment.

The protocol here described was registered in a public clinical trial registry (ClinicalTrials.gov Identifier: NCT05399043) and was developed following SPIRIT guidelines (http://www.spirit-statement.org/) and checklist [39]. Further details on SPIRIT schedule of enrolment, intervention, and assessment are provided in Fig 1.

### 2.1 Design, participants and procedures

**Fig 2:**
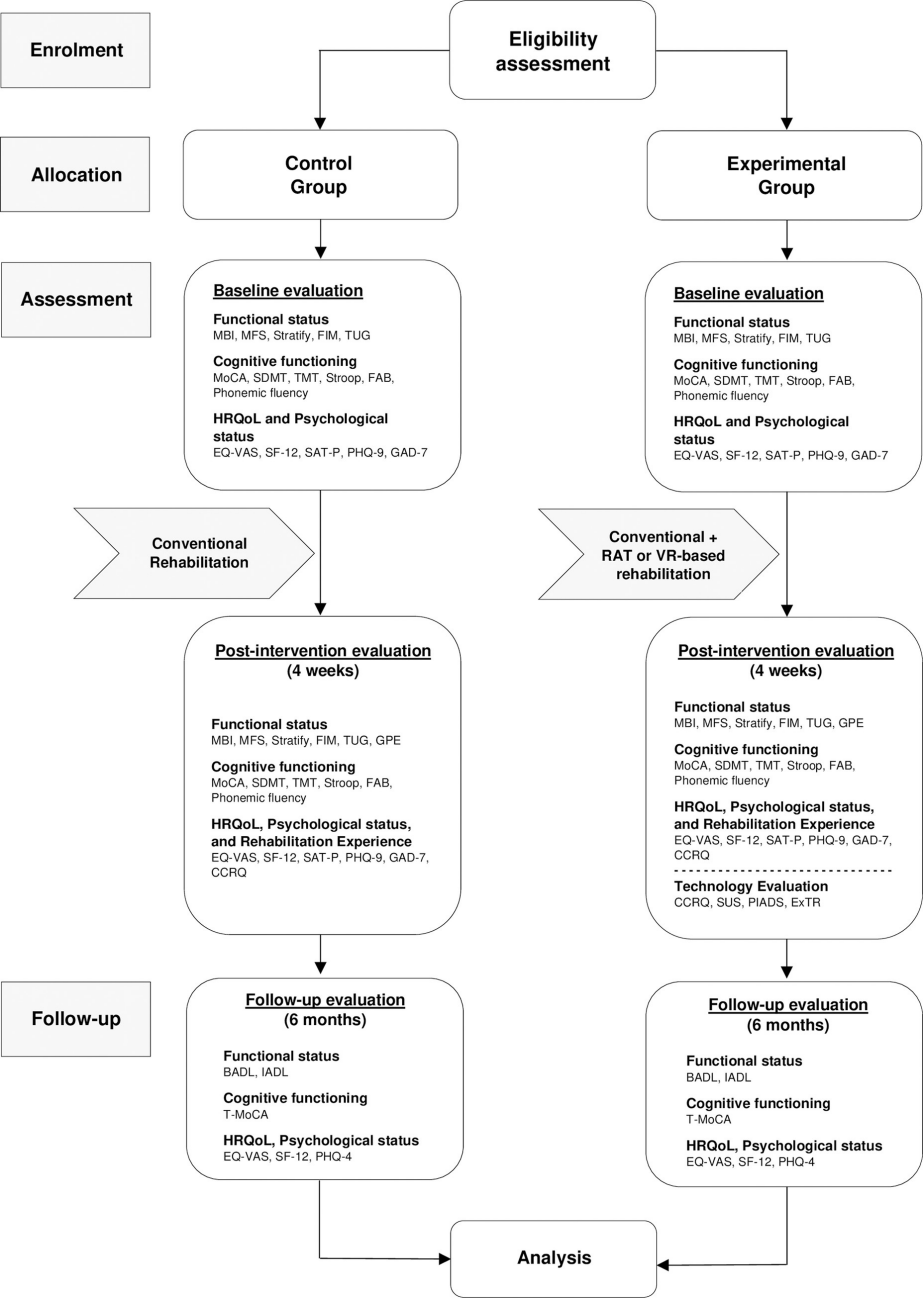
Study protocol flow chart. MBI, Modified Barthel Index; MFS, Morse Falls Scale; FIM, Functional Independence Measure; TUG, Timed Up & Go test; GPE, Global Perceived Effect; BADL, Basic Activities of Daily Living; IADL, Instrumental Activities of Daily Living; MoCA, Montreal Cognitive Assessment; SDMT, Symbol Digit Modalities Test; TMT, Trail Making Test; FAB, Frontal Assessment Battery; T-MoCA, Telephone Montreal Cognitive Assessment; EQ-VAS, Euro-QoL-VAS; SF-12, Short Form Health Survey-12; SAT-P, Satisfaction Profile; GAD-7, Generalized Anxiety Disorder-7; PHQ, Patient Health Questionnaire; CCRQ, Client-centred Rehabilitation Questionnaire; PIADS, Psychosocial Impact of Assistive Device Scale; SUS, System Usability Scale; ExTR, Experience in Technology-based Rehabilitation Schedule.

The protocol has a prospective, two-arm, open-label, non-randomized study design (Fig 2). In a real-world clinical setting, patients will be consecutively enrolled according to the following eligibility criteria: 18 years of age or older; diagnosis of acquired brain injury (ABI; e.g., TBI, stroke), Parkinson’s Disease (PD) or orthopaedic disease (i.e., total hip/knee arthroplasty) requiring rehabilitation intervention; absence of a severe clinical condition (i.e., chronic heart failure at class IV according to the New York Heart Association classification—NYHA-IV, ischemic heart disease at class IV according to the Canadian Cardiovascular Society classification—CCS-IV, neoplastic diseases, acute respiratory diseases); no severe cognitive impairment (screened with the Montreal Cognitive Assessment–MoCA ≤ 15.5) [40]; no language disorders (e.g., aphasia); absence of severe mental health condition or psychiatric disorder (evaluated according to the Diagnostic and Statistical Manual of Mental Disorders—DSM-V) potentially compromising participation in the study; absence or withdrawal of the informed consent to participate.

At hospital admission (or after clinical stabilization), patients will be screened according to the criteria described above by a panel consisting of physical and rehabilitation medicine specialists and researchers. Those considered eligible will undergo a multidimensional baseline evaluation including the functional status (i.e., disability, autonomy in the ADLs, risk of falls), cognitive functioning (i.e., attention, executive functions, reaction times, phonemic fluency), HRQoL, and psychological characteristics (i.e., quality of life satisfaction, anxiety and depression symptoms). After the intervention (4 weeks), patients will undergo the same evaluation battery to estimate pre- post-treatment differences and their rehabilitation experiences will be assessed. Participants will be divided into two subgroups, depending on their rehabilitation program. The first group will include patients who underwent conventional treatment exclusively, while the second group will be composed of those who participated in both conventional and RAT or VR-based rehabilitation. The latter, at post-intervention, will be further asked to evaluate the experience of use of the devices and the perceived usability (through a mixed-method approach), as well as the related perceived psychosocial impact. Technology evaluation will be also extended to the therapists involved in the rehabilitation programs. These will undergo mixed-method evaluation (i.e., questionnaire and semi-structured interview) aiming to collect data on the perceived usability of devices from the healthcare professional’s point of view. In conclusion, at six months following the rehabilitation program, all patients will be contacted by telephone and asked to provide information concerning their perceived functional status, level of autonomy in the ADLs, perceived HRQoL, and anxiety and depression symptoms.

### 2.2 Intervention and technological devices description

All patients will receive conventional treatment consisting of two daily one-hour sessions over 4 weeks, including traditional rehabilitation activities like range of motion exercises (passive, active assisted and active), progressive resistive exercises, balance and strength training, and aerobic conditioning. For the patients additionally participating in the RAT or VR-based rehabilitation, part of the duration of the whole treatment will be dedicated to the use of the devices, resulting in the same amount of rehabilitation for all participants. According to the technological device implemented (i.e., RAT or VR-based treatment), the intervention procedures and the related eligibility to participate will be checked according to the technology-specific guidelines and recommendations as appropriate [41–43]. The intervention will be delivered following the routine multidisciplinary clinical practice of the Institution where the study will be conducted [44]. Specifically, for each patient, the clinical care pathway will be based on the integration of the International Classification of Diseases (ICD) and the International Classification of Functioning Disability and Health (ICF) models as recommended by the WHO framework [45]. Therefore, adopting a biopsychosocial approach, the rehabilitation procedures will be adapted according to the patient’s medical diagnosis, disability severity, and rehabilitation objectives in favor of an individualized rehabilitation project and program. Therefore, if necessary, patients may undergo parallel rehabilitation activities that will involve different healthcare professionals (i.e., occupational therapists, speech therapists, psychologists).

Technology-based rehabilitation will be delivered through the following exoskeleton or non-immersive VR devices.

#### Lokomat®

The Lokomat (Hocoma AG, Switzerland) is a commercially available device consisting of a motorized exoskeleton facilitating symmetric gait patterns by the integration of computer-controlled linear actuators on patients’ hip and knee joints and a body-weight support treadmill system (BWTS). The device also provides pace and driving force adjustment according to each patient’s individual needs, as well as hip and knee stiffness and isometric strength monitoring. Increased feedback through a screen displaying non-immersive VR is supplied.

#### Armeo-Spring®

The Armeo-Spring (Hocoma AG, Switzerland) is a commercially available exoskeleton device for integrated arm and hand therapy. It is a three-dimensional, passive, exoskeleton robot that provides gravity compensation, offsetting the device and the patient’s upper limb with the help of a spring in place of robotic actuators. A screen displaying game-based VR environments to interact with during the therapy session is also integrated.

#### ProKin 252

The ProKin (TecnoBody SRL, Italy) is a proprioceptive-stabilometric assessment and training machine. It consists of a mobile platform including an electro-pneumatic system that employs an electronic pressure regulator to allow for independent stability control on the two axes of antero-posterior and lateral movements. The joint motion data, collected in the kinesthetic trace, is displayed on an integrated monitor that simultaneously provides game-based non-immersive VR exercises. The system can therefore provide sensory-motor monitoring along with both static and dynamic (monopodalic or bipodalic) balance training with real-time biofeedback.

#### D-Wall

The D-Wall (TecnoBody SRL, Italy) is a digital mirror device consisting of a strength platform paired to a control PC and a high-resolution 3D Kinect camera equipped with infrared rays that allows for motion-capture. The system provides immediate feedback of a patient’s movements on a large LCD monitor delivering VR-based mirror therapy and game-based exercises. The software includes different programs with different difficulties that can provide specific non-immersive VR-based recovery programs for ankle, lower limbs, trunk, shoulder, and upper limb rehabilitation.

#### Walker View

The Walker View (TecnoBody SRL, Italy) consists of a treadmill equipped with a control PC, a large LCD monitor, and an integrated 3D Kinect camera for motion capture. The detection load surface system of the treadmill, together with the motion-capture device, makes it possible to collect data on a patient’s balance and motion. Through the monitor, the patient is provided with biofeedback on gait analysis and non-immersive VEs to interact with during the training. Moreover, the device includes a body-weight support system ensuring locomotion therapy for patients at high risk of falls.

#### Riablo^TM^

The Riablo (CoRehab, Italy) is a medical device consisting of wearable sensors to don on patients’ limbs, and a stabilometric platform that transmits motion data to a software that provides biofeedback and displays non-immersive VR games through a screen. The system makes it possible to monitor and train a patient’s limb range of motion, balance, and anterior-posterior and lateral flexion and extension of the spine with the sensor placeable in the lumbar area. The device was designed to address orthopedic and neuromotor conditions to facilitate passive and active mobility and proprioceptive capacity recovery.

### 2.3 Data collection

Following preliminary collection of the socio-demographic (i.e., age, gender, living conditions, marital status, occupation, caregiver identification, and education) and clinical (i.e., BMI, primary diagnosis, comorbidities, health-related risk factors, and COVID-19-related anamnesis) characteristics, patients will undergo a multidimensional evaluation. This will include the functional status, cognitive functioning, perceived HRQoL and psychological aspects. All patients will be also asked to report any prior experience in using technological devices during rehabilitation. Moreover, the same information will be collected from the therapists involved, along with their individual socio-demographic and occupation-related data (i.e., age, gender, occupation, and overall seniority).

#### 2.3.1 Functional status

*Modified Barthel Index (MBI)*. The MBI is a 100-point rating scale measuring a patient’s ability to perform 10 different types of ADLs (i.e., chair/bed transfer, ambulation, stair climbing, toilet transfer, bowel control, bladder control, bathing, dressing, personal hygiene, feeding). Each activity is assigned a numeric value in accordance with the patient’s assistance needs. Lower scores indicate less independence, whereas higher scores reflect greater independence [46]. This scale has been widely used in the hospital setting and, compared to the original version (the Barthel Index scale–BI), it has been shown to be test-retest reliable in the rehabilitation context [47].

*Morse Fall Scale (MFS)*. The MFS is a reliable and validated tool to measure patient’s risk of falls. It consists of six items exploring: the patient’s history of falls (immediate or within 3 months), any secondary diagnosis, any need for ambulatory assistance, if the patient receives intravenous therapy, the patient’s gait and ability to transfer, and the mental status (orientation). The total score ranges from 0 to 125 with higher scores reflecting a greater risk of falling (0–24: no risk; 25–44: low risk; ≥45: high risk) [48].

*Stratify Scale*. The stratify Scale is a validated 5-point tool measuring patient’s risk of falls. For the current study, this instrument will be administered to those patients with a higher risk of falling (MFS ≥ 45). It comprises five items providing further insight on the following risk factors: past history of falling, patient agitation, visual impairment, incontinence, transfer and mobility. From the original validation, a predictive cut-off score of risk of falling was fixed at ≥ 2 points [49].

*Functional Independence Measure (FIM)*. The FIM is a widely used and reliable functional assessment tool generally adopted in rehabilitation as a functional outcome indicator. It evaluates a patient’s disability level indicating the degree of assistance required in the ADLs. It comprises 18 items. Of these, 13 consider motor and 5 cognitive domains. All items are measured on a 7-point Likert scale with a total score ranging from 18 (complete dependence) to 126 (complete independence). Two sub-scores can be calculated: FIM motor (range: 13–91) and FIM cognitive (range: 5–35) [50, 51].

*Timed Up & Go Test (TUG)*. The TUG is a functional test measuring a patient’s risk of falling, as well as static and dynamic balance. Specifically, it measures the time a patient takes to stand up from an armchair, walk forward 3 m, turn, walk back to the chair, and sit down. The longer the time this takes, the higher the risk of falling, evaluated with a cut-off score that varies according to the clinical population [52].

*Basic Activities of Daily Living (BADL)*. The BADL is a rating scale assessing a patient’s level of independence in the basic activities in relation to the daily environment (i.e., bathing, dressing, toilet, continence, transferring, feeding). It comprises a total of six items scored on a 3-point scale. Higher scores indicate stronger independence in basic ADLs [53].

*Instrumental Activities of Daily Living (IADL)*. The IADL provides an assessment of a person’s level of independence in performing more complex and instrumental activities (i.e., using the telephone, making purchases, cooking, housekeeping, doing laundry, handling money, using means of transport, responsibility for his/her own medications). Higher scores reflect stronger autonomy in more complex ADLs [54].

*Global Perceived Effect (GPE)*. A single item evaluating the perceived efficacy of the treatment will be administered at the end of the intervention. Patients will be asked to rate, on a scale ranging from 1 to 7, how much their condition has improved or deteriorated following the treatment they received. To check item responsiveness, the same numerical scale will also be administered to the therapists involved to evaluate how much patients’ conditions have changed following the intervention provided [55].

#### 2.3.2 Cognitive functioning

Montreal Cognitive Assessment (MoCA)

The MoCA is a widely used screening test covering eight cognitive domains: visuospatial abilities, executive functions, attention, concentration, short-term and delayed verbal memory, working memory, language, and orientation to time and space. This test has shown satisfactory psychometric properties including high sensitivity and specificity to identify cognitive impairment in a wide array of diseases [40]. The pre- post-intervention change will be estimated by administering the alternate validated form of the test [56]. Moreover, the telephone-based version of the MoCA (T-MoCA) will be administered at the 6-month follow-up [57].

*Symbol Digit Modalities Test (SDMT)*, *Oral version*. The SDMT measures speeded information processing by evaluating a patient’s attention, perceptual and motor speed, and visual scanning. The patient is given a sheet with nine symbols, each paired with a number on top of the page. A randomized and sequential assortment of these symbols are presented on the remainder of the page. Within 90 seconds, the patient is asked to scan each symbol and verbally respond with the corresponding number [58].

Trail Making Test (TMT)

The TMT comprises two parts (TMT-A and TMT-B). The TMT-A consists of a standardized page with scattered numbers (from 1 to 25) within circles. The patient is asked to connect the numbers in ascending order as quickly as possible. Similarly, the TMT-B presents a standardized page including numbers (from 1 to 13) and letters (from A to N). The patient is instructed to connect the numbers and the letters in order and alternate them. While the TMT-A evaluates psychomotor speed and visual search attention skills (i.e., visual scanning, graphomotor speed, and visuomotor processing speed), the TMT-B measures executive function components (i.e., working memory, inhibition control, or set-switching abilities) [59].

*Stroop colour word test*. For the present study protocol, a shortened version of the Stroop test is used [60]. This tool consists of three parts measuring selective attention, cognitive flexibility and inhibition, and sensitivity to interference. In the first part, the patient is given a list of color words (i.e., red, blue, green) and is asked to read them as quickly as possible. In the second part, the same colors are presented as colored circles. Here, the patient is asked to name them. In the last part, a list of the same color words is presented printed in nonmatching colors. The patient is instructed to name the colors disregarding the verbal content of the words. The final score is obtained by computing the time interference effect (based execution time) and the error interference effect (based on number of errors).

Frontal Assessment Battery (FAB)

The FAB is a brief battery of six cognitive tasks to assess executive functions (i.e., conceptualization and abstract reasoning, lexical verbal fluency and mental flexibility, motor programming and executive control of action, self-regulation, resistance to interference, inhibitor control, and environmental autonomy). Each task is evaluated with a specific subtest. A total score of 18 points is obtainable, with higher scores indicating better performance [61].

Phonemic verbal fluency test

To evaluate phonemic fluency, a task-specific test belonging to a wider battery that measures cognitive deterioration will be implemented [62]. In this test, the patient is asked, in three different trials (each lasting 1 minute), to generate as many words as possible beginning with the letters “F”, “A”, and “S”. For each word generated, one point will be assigned. The higher the final score, the better the performance on the cognitive test.

#### 2.3.3 HRQoL and psychological status

EuroQoL-VAS (EQ-VAS)

The EQ-VAS is a widely used, reliable tool to quantitatively record a patient’s self-rated health. The patient is asked to respond on a vertical visual analogue scale (0 = the worst health you can imagine; 100 = the best health you can imagine) [63].

Short Form Health Survey-12 (SF-12)

The SF-12 evaluates functional health and well-being according to the patient’s perspective. It comprises 12 items measuring different health-related domains (i.e., physical functioning, general health, role physical, role emotional, vitality, bodily pain, social functioning, and mental health) generating two aggregate summary indexes: the physical component summary (PCS) score and the mental component summary (MCS) score, indicating physical health and mental health, respectively. Higher total scores indicate better health [64].

Generalized Anxiety Disorder-7 (GAD-7)

The GAD-7 is a validated questionnaire measuring the severity of anxiety symptoms in the previous two weeks. It is composed of 7 items scored on a 4-point Likert scale (0 = not at all; 3 = nearly every day). Higher scores indicate more severe anxiety symptomatology. Scores of 5, 10, and 15 represent the cut-offs for mild, moderate, and severe anxiety, respectively [65].

Patient Health Questionnaire-9 (PHQ-9)

The PHQ-9 is a validated questionnaire evaluating the severity of depression symptoms in the previous 2 weeks. It consists of 9 items scored on a 4-point Likert scale (0 = not at all; 3 = nearly every day). Higher scores indicate more severe depression symptomatology. Scores of 5, 10, 15, and 20 represent the cut-offs for mild, moderate, moderately severe, and severe depression, respectively [66]. A 4-item shortened version (PHQ-4) was developed, including two items of the GAD-7 and two of the PHQ-9, as a screening measure for both anxiety and depression symptomatology. This version will be implemented at the telephone-based 6-month follow-up [67].

*The Satisfaction-Profile (SAT-P)*. The SAT-P is a validated instrument to evaluate patient satisfaction in the last month with different domains associated with HRQoL. It is composed of 32 items evaluated on a 10-cm horizontal visual analogue scale ranging from “extremely dissatisfied” to “extremely satisfied”. For the present study protocol, three items will be included in the evaluation, specifically measuring personal satisfaction with mood, resistance to physical fatigue, and mental efficiency [68].

*Client-Centred Rehabilitation Questionnaire (CCRQ)*. The CCRQ is a validated questionnaire measuring a patient’s subjective experience of care in rehabilitation settings. It consists of 33 items scored on a 5-point Likert scale (1 = strongly agree; 5 = strongly disagree) and grouped into seven subscales (i.e., patient participation in decision making and goal setting, patient-centred education, evaluation of outcomes from the patient’s perspective, family involvement, emotional support, co-ordination/continuity, physical comfort). Reversed scoring is applied with higher scores reflecting a better rehabilitation experience [69].

#### 2.3.4 Technology evaluation

For the patients also participating in the technology-based rehabilitation program, the following additional questionnaires will be administered.

*Psychosocial Impact of Assistive Device Scale (PIADS)*. The PIADS is a checklist of 26 self-report items, presented as a list of common words or short sentences. It comprises three subscales (i.e., adaptability, competence, and self-esteem) evaluating the degree of acceptance related to the device as well as its impact on different domains of a patient’s subjectivity. Patients are asked to rate their degree of perceived satisfaction or improvement related to each item on a 7-point Likert scale. The score is meant to be either positive (+1, +2, +3 scores) or negative (-1, -2, -3 scores), with a central tendency marked as zero that indicates no perceived change after using the device. Higher scores reflect the technological device’s stronger psychosocial impact [70].

*System Usability Scale (SUS)*. The SUS is a widely used questionnaire to evaluate the perceived usability of systems and devices. It consists of 10 items measuring a device’s ease-of-use and learnability. For each item, the patient is asked to respond on a 5-point Likert scale (0 = strongly disagree; 4 = strongly agree). Total scores are converted into a percentile ranking (0–100) with higher scores indicating higher usability [71]. For the present study protocol, this tool will be administered to both patients and therapists. In addition, therapists will undergo a semi-structured interview aiming to collect qualitative data on the perceived usability of devices from a healthcare professional’s perspective. This data will be integrated to the scale.

*Experience in Technology-based Rehabilitation Schedule (ExTR)*. Based on a preliminary literature review [72], a schedule to evaluate patients’ experience of use of the devices in the rehabilitation program was developed. The ExTR aims to provide mixed-method information concerning different domains related to patient’s subjective experience of use (i.e., learnability, acceptability, usefulness, adaptability, adverse effects, engagement, enjoyment, safety, perceived effectiveness). The schedule includes 15 items scored on a scale ranging from 0 (not at all) to 4 (extremely). Higher scores indicate better experience of use. To provide further insight into the overall experience of use, an additional open-ended question was added at the end of the tool to elicit patients’ socio-cognitive and experiential factors related to the technology-based program (e.g., emotions, expectations, motivation, satisfaction).

### 2.4 Data management

Data collection is currently ongoing. The data collected throughout the study phases will be handled and stored in accordance with the General Data Protection Regulation (GDPR) 2018. The use of the study data will be controlled by the principal investigator. All data and documentations related to the study will be stored in accordance with applicable regulatory requirements and their access will be restricted to authorized personnel.

### 2.5 Ethics considerations

The current study protocol (PHTinRehab Study) was approved by the Ethics Committee of ICS Maugeri, Pavia Italy (February 2021, protocol n. 2517CE). All patients will be given the informed written consent to join the study, and asked to sign it. Their participation will be voluntary and will not affect the healthcare process. The entire study will be conducted in accordance with the Declaration of Helsinki and all relevant guidelines and regulations covering respect for the rights and dignity of participants.

### 2.6 Statistical analyses

Descriptive statistics will be conducted on participants’ socio-demographic and clinical characteristics, functional status, cognitive functioning, and HRQoL, and psychological status. Explorative analyses will be then conducted to estimate pre- post-treatment changes and long-term effects concerning the multidimensional evaluation performed. Particularly, these will be carried out by comparing patients who share the same medical disease and who experienced the use of the same technological device during rehabilitation. Within- and between-group pre- post-intervention differences will be tested with the paired-samples *t*-test and independent samples *t*-test, respectively. Additionally, a repeated-measures ANOVA test will be used to assess the longitudinal trajectories. Regarding technology evaluation, descriptive statistics will be conducted on devices usability, experience of use and psychosocial impact. Furthermore, post-hoc analyses will be performed to estimate the differences on technology evaluation in relation to the clinical condition and severity of participants. As for therapists’ devices experience of use, the semi-structured interviews will be analysed through inductive thematic analysis [73]. In conclusion, association analyses (e.g., correlations, regressions) will be carried out to explore the inter-relationships among all the variables measured. The Statistical Package for the Social Sciences (SPSS, version 28.0) will be implemented for data analyses. Sample size adequacy was estimated by resorting to power analysis [74], using G*Power Software Version 3.1.9.7 [75] on a repeated-measures ANOVA (within-between interaction) with three measurements, two groups, and a correlation among repeated measures of 0.5 (power: 0.80; α = 0.05). The required sample size to detect small-medium effect sizes (f = 0.20) was a minimum of 42 patients.

## 3. Discussion

The present work aimed to describe a study protocol exploring the short- and long-term perceived biopsychosocial effects and the experience of use of robotic and VR devices in patients undergoing neuromotor rehabilitation. For this purpose, a prospective multidimensional evaluation investigating the patient’s functional status (i.e., disability, autonomy in the ADL, risk of falls), cognitive functioning (i.e., attention and executive functions), perceived HRQoL, and psychological status (i.e., anxiety and depression symptoms) was outlined. Besides, the psychosocial impact of the technological devices implemented will be explored and an evaluation of the perception of their usability and experience of use will be carried out by considering both patients’ and therapists’ perspectives, through a mixed-methods approach.

Based on the aims of this study protocol and according to the existing literature, it is expected that the present contribution may corroborate the evidence of prior works on patient’s motor recovery highlighting the added value of integrating robotics and VR in neuromotor rehabilitation programs. Most importantly, the development of the current study protocol was aimed at giving more centrality to the psychological dimension of patient’s health, contributing overall to deepen knowledge on the short- and long-term effectiveness of RAT and VR-based rehabilitation beyond motor improvement. Furthermore, the mixed-methods investigation of the perception of the technology implemented, in terms of experience of use and perceived usability, not only will shed light on devices’ technical strengths and limitations with a view to improved technology deployment, but it will also provide crucial evidence into patient’s attitude towards technology-based rehabilitation, including therapy engagement, adherence, and efficacy.

So far, diverse clinical trials protocols have been recently developed with the purpose of estimating the motor and functional benefits of RAT and VR-based therapy in different clinical populations [76–83]. Nevertheless, it must be noted that for the most, although factors like patient’s mental status or technology acceptance were investigated, these were considered as secondary outcomes essentially. The added value of the present protocol lies therefore in the attempt to better understand the psychological impact and the experience of use of technological devices in neuromotor rehabilitation, thereby providing a deeper knowledge and discussion, net of patient’s motor and functional changes.

### 3.1 Limitations

The present study protocol presents some design-related limitations. The first limit is the absence of randomization. However, although randomized control trials represent essential research tools with strong internal validity, it must be recognized that they may suffer from low generalizability to real life conditions [84]. For this reason, the present study protocol was outlined within a real-world clinical setting, where the actual effectiveness of technology will be explored [85]. Another limitation is the single-center design and the limited sample size calculated. Regarding the latter, even reaching the required sample size, the evidence from the reports that will be generated will not be sufficient to draw generalizable conclusions and should be considered as preliminary. Accordingly, there is a need for multicenter and large-scale studies in order to generalize the benefits of RAT and VR-based rehabilitation particularly on outcomes that are not strictly related to motor functioning. In conclusion, the use of self-report evaluation may be limiting for different reasons, including the risk of false-positive, lack of sensitivity to change, and social desirability bias. Nonetheless, this evaluation approach was preferred given some advantages like good clinical and research applicability, high practicality of use, and satisfactory cost-effectiveness.

### 3.2 Implications

Relevant practical implications should be noted. The study protocol described was developed adopting a multidimensional and multidisciplinary approach. This choice comes from the intention to put the study into a biopsychosocial framework, where multiple patients’ health-related domains, and their complementarity, are considered throughout technology-based rehabilitation programs [86]. This approach may help to expand the perspective on patient recovery within this ever-growing intervention procedure, consequently providing a deeper understanding on the definition of more tailored recovery pathways. Following this line, also the involvement of different pathologies with different degrees of severity, as well as the use of different technological devices will contribute to obtaining more heterogenous findings and deeper knowledge on how to improve technology implementation and its effectiveness. In conclusion, alongside the continuously aging population and the related increase in chronic diseases and multimorbidity, providing more complex intervention guidelines along with optimal and tailored recovery has become of paramount concern [87]. Accordingly, high technology may represent a valuable tool to respond to the emerging public health challenges and, more broadly, a practical solution for healthcare sustainability issues.

## Supporting information

S1 Appendix SPIRIT guidelines checklist. (PDF)

S2 Appendix Copy of the protocol approved by the ethics committee. (PDF)
