# Supplementary material for: Biopsychosocial effects and experience of use of robotic and virtual reality devices in neuromotor rehabilitation: A study protocol
Source: PLoS One. 2023 Mar 10;18(3):e0282925. doi: 10.1371/journal.pone.0282925 (PMC10004562; doi:10.1371/journal.pone.0282925)
Supplement: S2 Appendix — (PDF) [file pone.0282925.s002.pdf]

Perception of High Technology in Rehabilitation: a prospective real-life study on usability, effectiveness, and health-related quality of life. PHTinRehab.

**Rationale**	Over the past two decades, the aging population and the related increase of acute and chronic diseases have led to the need for urgent healthcare solutions, including the implementation of interdisciplinary and innovative approaches in patients' care. Contextually, the field of neuromotor rehabilitation has shown deep interest in the deployment of robotic and virtual reality (VR) devices in recovery programs, given their multipurpose application.<br><br>Robot-assisted therapy (RAT) has reported promising evidence so far, showing advantages like the possibility to provide repetitive, intensive, and task-oriented rehabilitation activities, including the opportunity to implement smaller workforce, optimized exercise, and real-time quantitative motor assessment and monitoring. To date, diverse robot typologies (ie, exoskeletons, end-effectors, soft-robots) have been implemented to treat different chronic and complex diseases like acquired brain injury (ABI), spinal cord injury (SCI), multiple sclerosis (MS), and Parkinson's disease (PD) reporting evidence in favor of their feasibility and an improvement in patient functionality, autonomy and health-related quality of life (HRQoL).<br>Likewise, VR has been shown to be a promising tool to enhance rehabilitation outcomes. When various technical devices (eg, head-mounted displays, motion capture and tracking systems) are implemented, it can deliver realistic experiences by interacting with virtual environments (VEs) closely resembling everyday environments. To date, plenty of studies have tested and demonstrated the efficacy, for example, of VR-treadmill trainings for lower limbs, to improve gait and balance, ultimately reducing risk of falls, or of exercises in reaching and grasping virtual objects to target arms and manual movement, dexterity, and coordination. Besides, thanks to the multimodal and multisensory stimulation it can provides, VR has revealed to be an effective tool to obtain significant changes in cognitive outcomes (eg, memory, visual attention, executive functions). Lastly, such stimulation has been shown to have potential for increased patient engagement in the rehabilitation program, ultimately improving treatment compliance.<br><br>Nevertheless, alongside the technological devices' complexity and the related clinical application, ensuring adequate patient engagement and adherence to treatment still represents an open challenge. Accordingly, the introduction of technology in rehabilitation programs has raised the issue of devices usability and experience of use. Usability specifically refers to a patient's perception and ability to use a device effectively, efficiently, and satisfactorily. Factors like device ease-of-use and learnability are therefore essential when aiming to achieve adequate technology acceptance rate. Such factors, however, do not always sufficiently explain the complexity of the experience of use. To better expand the concept of device use, socio-cognitive and experiential factors (eg, emotions, motivation, satisfaction) need to be considered too.<br><br>In light of the data in the literature, beyond motor and functional recovery what has been scarcely addressed up to date is the potential of RAT and VR to improve patient's psychosocial functioning. Moreover, to optimally enhance technological devices deployment in rehabilitation programs, a deeper understanding on their experience of use is increasingly needed.

**Aims**	Based on the existing literature, the purpose of this study protocol is to explore, in a real-world clinical setting, both the experience of use and the biopsychosocial effectiveness of robotic and VR technology in patients undergoing neuromotor rehabilitation. In particular, this protocol outlines the procedures for exploring the perception and the effects of such technology across different neurological (ie, ABI, PD) and orthopedic (ie, total knee/hip arthroplasty) conditions and the implicit differences of the technological devices implemented. Accordingly, within/between-groups observations will be made as appropriate by considering consistent clinical populations and intervention procedures.<br>Specifically, the study objectives are:<br>(a) to explore pre- post-treatment differences and long-term (6-month follow-up) effects concerning: patient's HRQoL, quality of life satisfaction, anxiety and depression symptoms, cognitive functioning, and functional status;<br>(b) to measure patient's perceived experience of the rehabilitation program, as well as the perceived experience of using the robotic and VR devices (user experience and device usability) and the related psychosocial impact;<br>(c) to observe the differences in terms of device experience of use in relation to the pathology, disease severity, and the type of device implemented;<br>(d) to extend the evaluation of the device's perceived usability to the therapists and explore to what extent this is associated to patient's perception of device usability and rehabilitation experience.
**Study design**	Prospective, two-arm, parallel, open-label non-randomized trial
**Eligibility Criteria**	<u>Inclusion criteria</u><br>• 18 years of age or older;<br>• Diagnosis of acquired brain injury (ABI) or Parkinson's Disease (PD) or total knee/hip arthroplasty, requiring rehabilitation intervention.<br><br><u>Exclusion criteria</u><br>• severe clinical condition (ie, chronic heart failure [New York Heart Association Classification-IV - NYHA-IV], ischemic heart disease [Canadian Cardiovascular Society Classification-IV - CCS-IV], neoplastic disease, acute respiratory disease);<br>• severe cognitive impairment (MoCA ≤ 15.5);<br>• language deficits;<br>• severe mental health condition or psychiatric disorder compromising participation in the study;<br>• absence or withdrawal of the informed consent to participate.
**Intervention**	For each patient, the clinical care pathway will be based on the integration of the International Classification of Diseases (ICD) and the International Classification of Functioning Disability and Health (ICF) models as recommended by the WHO framework. Therefore, through the adoption of a biopsychosocial approach, the rehabilitation procedures will be adapted according to the patient’s medical diagnosis, disability severity, and rehabilitation objectives in favor of an individualized rehabilitation project and program. Depending on the rehabilitation program, participants will be therefore divided a posteriori into two subgroups.

	<u>Group 1 Multidisciplinary Conventional Rehabilitation</u><br><br>Patients will receive conventional treatment consisting of two daily one-hour sessions (4 weeks) and including traditional rehabilitation activities like range of motion exercises (passive, active assisted and active), progressive resistive exercises, balance and strength training, and aerobic conditioning. The intervention procedures will follow the routine multidisciplinary clinical practice of the Institute where the study will be carried out. If necessary, patients might therefore undergo parallel rehabilitation activities (ie, occupational therapy, speech therapy, psychological support).<br><br><u>Group 2 Multidisciplinary Conventional Rehabilitation plus technology-based treatment</u><br><br>According to their individualized rehabilitation project, patients will undergo RAT or non-immersive VR-based rehabilitation.<br><br>RAT will consist of three times a week one-hour session (4 weeks) and will be conducted by the implementation of an exoskeleton device for the upper limbs (Armeo-Spring™) or for the lower limbs (Lokomat™).<br><br>VR-based rehabilitation will consist of one daily one-hour session (4 weeks) and will be conducted by the implementation of a single non-immersive VR device among the Riablo™ (Corehab, Italy), the ProKin 252™ (TechnoBody SRL, Italy), the D-Wall™ (TechnoBody SRL, Italy), the Walker View™ (TechnoBody SRL, Italy).<br><br>Part of the duration of the whole treatment will be dedicated to the use of the device, resulting in the same amount of rehabilitation for all participants.
**Evaluation**	At hospital admission (or after clinical stabilization), all eligible patients will undergo a multidimensional baseline evaluation including the functional status, cognitive functioning, HRQoL, and psychological status.<br><br>After the intervention (4 weeks), patients will undergo the same evaluation battery to estimate pre- post-treatment differences and their rehabilitation experiences will be assessed. At post-intervention, Group 2 will be further asked to evaluate the experience of use of the devices and the perceived usability (through a mixed-method approach), as well as the related perceived psychosocial impact. Technology evaluation will be extended to the therapists involved. These will undergo a mixed-methods evaluation (ie, questionnaire and semi-structured interview) on devices' experience of use and usability.<br><br>At six months following the rehabilitation program, all patients will be contacted by telephone and asked to provide information concerning their perceived functional status, level of autonomy in the ADLs, perceived HRQoL, and anxiety and depression symptoms.
**Outcome measures**	Preliminary collection of the socio-demographic (i.e., age, gender, living conditions, marital status, occupation, caregiver identification, and education) and clinical (i.e., BMI, primary diagnosis, comorbidities, health-related risk factors, and COVID-19-related anamnesis) characteristics will be conducted for all patients. Of the therapists involved, individual socio-demographic and occupation-related data (i.e., age, gender, occupation, and overall seniority) will be also collected.<br><br><u>Functional Status Evaluation</u><br><br>• Modified Barthel Index (MBI);<br>• Functional Independence Measure (FIM);<br>• Morse Fall Scale (MFS);<br>• Stratify Scale;<br>• Timed Up and Go Test (TUG);<br>• Basic Activities of Daily Living (BADL);<br>• Instrumental Activities of Daily Living (IADL).

	<u>Cognitive Functioning Evaluation</u><br>• Montreal Cognitive Assessment (MoCA);<br>• Frontal Assessment Battery (FAB);<br>• Trail Making Test (TMT);<br>• Phonemic Fluency;<br>• Stroop Test;<br>• Symbol Digit Modalities Test (SDMT).<br><br><u>HRQoL and psychological Status Evaluation</u><br>• EuroQoL-VAS (EQ-VAS);<br>• Short Form Health Survey-12 (SF-12);<br>• The Generalized Anxiety Disorder (GAD-7);<br>• Patient Health Questionnaire (PHQ-9, PHQ-4);<br>• The Satisfaction-Profile (SAT-P);<br>• Client-Centred Rehabilitation Questionnaire (CCRQ);<br>• Psychosocial Impact of Assistive Device Scale (PIADS);<br>• System Usability Scale (SUS);<br>• Experience in Technology-based Rehabilitation Schedule (ExTR).
**Data analyses**	Pre- post-intervention differences and long-term trajectories will be carried out by comparing patients who share the same medical disease and who experienced the use of the same technological device during rehabilitation. Within- and between-group pre-post-treatment changes will be tested with the paired-samples t-test and independent samples t-test, respectively. Repeated-measures ANOVA test will be used to assess the longitudinal effects. Regarding technology evaluation, descriptive statistics will be conducted on devices usability, experience of use and psychosocial impact. Then, post-hoc analyses will be performed to estimate the differences in relation to the clinical condition and severity of participants. The semi-structured interviews will be analysed through inductive thematic analysis. In conclusion, association analyses (e.g., correlations, regressions) will be carried out to explore the inter-relationships among all the variables measured.<br><br><u>Sample Size</u><br>Sample size adequacy was estimated on a repeated-measures ANOVA (within-between interaction) with three measurements, two groups, and a correlation among repeated measures of 0.5 (power: 0.80; $\alpha = 0.05$). The required sample size to detect small-medium effect sizes ($f = 0.20$) was a minimum of 42 patients.
